# The Chromosome-Level Genome of Miracle Fruit (*Synsepalum dulcificum*) Provides New Insights Into the Evolution and Function of Miraculin

**DOI:** 10.3389/fpls.2021.804662

**Published:** 2022-01-03

**Authors:** Zhuang Yang, Zhenhuan Liu, Hang Xu, Yayu Chen, Pengmeng Du, Ping Li, Wenjie Lai, Haiyan Hu, Jie Luo, Yuanhao Ding

**Affiliations:** ^1^Hainan Key Laboratory for Sustainable Utilization of Tropical Bioresource, College of Tropical Crops, Hainan University, Haikou, China; ^2^Sanya Nanfan Research Institute of Hainan University, Hainan Yazhou Bay Seed Laboratory, Sanya, China

**Keywords:** genome evolution, miraculin, gene evolution, *Synsepalum dulcificum*, gene function

## Abstract

Miracle fruit (*Synsepalum dulcificum*) is a rare valuable tropical plant famous for a miraculous sweetening glycoprotein, miraculin, which can modify sour flavors to sweet flavors tasted by humans. Here, we present a chromosome-level high-quality genome of *S. dulcificum* with an assembly genome size of ∼550 Mb, contig N50 of ∼14.14 Mb, and 37,911 annotated protein-coding genes. Phylogenetic analysis revealed that *S. dulcificum* was most closely related to *Camellia sinensis* and *Diospyros oleifera*, and that *S. dulcificum* diverged from the *Diospyros* genus ∼75.8 million years ago (MYA), and that *C. sinensis* diverged from *Synsepalum* ∼63.5 MYA. Ks assessment and collinearity analysis with *S. dulcificum* and other species suggested that a whole-genome duplication (WGD) event occurred in *S. dulcificum* and that there was good collinearity between *S. dulcificum* and *Vitis vinifera*. On the other hand, transcriptome and metabolism analysis with six tissues containing three developmental stages of fleshes and seeds of miracle fruit revealed that Gene Ontology (GO) terms and metabolic pathways of “cellular response to chitin,” “plant–pathogen interaction,” and “plant hormone signal transduction” were significantly enriched during fruit development. Interestingly, the expression of miraculin (Chr10G0299340) progressively increased from vegetative organs to reproductive organs and reached an incredible level in mature fruit flesh, with an fragments per kilobase of transcript per million (FPKM) value of ∼113,515, which was the most highly expressed gene among all detected genes. Combining the unique signal peptide and the presence of the histidine-30 residue together composed the main potential factors impacting miraculin’s unique properties in *S. dulcificum*. Furthermore, integrated analysis of weighted gene coexpression network analysis (WGCNA), enrichment and metabolite correlation suggested that miraculin plays potential roles in regulating plant growth, seed germination and maturation, resisting pathogen infection, and environmental pressure. In summary, valuable genomic, transcriptomic, and metabolic resources provided in this study will promote the utilization of *S. dulcificum* and in-depth research on species in the Sapotaceae family.

## Introduction

Miracle fruit (*Synsepalum dulcificum*) is famous for a sweetening glycoprotein, namely, miraculin, which has a taste-modifying activity that converts sour taste to sweet (Kurihara and Beidler, 1968; Misaka, 2013; Niu et al., 2020). Miracle fruit is a native plant of tropical west and central Africa, occasionally distributed from West Africa to the Congo (Achigan-Dako et al., 2015). In the 1960s, miracle fruit was introduced to China as the national gift from the Republic of Ghana to Premier Enlai Zhou. Exports of miracle fruit are protected and prohibited in China and West Africa due to its high prize. Miracle fruit is an evergreen shrub belonging to the *Synsepalum* genus of the Sapotaceae family that has ∼50 genera and 1,100 species. Most species in Sapotaceae are trees and shrubs, which commonly have unique fruit flavors and are widely distributed in tropical and subtropical regions (Khayi et al., 2020).

The height of miracle fruit trees is ∼1.5–4.5 m, with many branches, dense leaves, and small white flowers, which usually flower from February to May and ripen in March, June, and October. The fruit is an ovoid to oblong berry, ∼2 cm long and 1 cm wide, with white and juicy flesh (Tchokponhoué et al., 2021). When the fruit matures, the color of the peel turns an attractive bright red from green in a relatively short time. Miracle fruit can be both autogamic and allogamic, commonly pollinated by insects such as ants, and mainly propagated through seeds (Tchokponhoué et al., 2019a,b; Akinmoladun et al., 2020).

Due to its dramatic taste-modifying activity, miraculin is usually used as a sweetening additive in the beverage and food industry and as an adjuvant for treating diabetic patients with insulin resistance (Chen et al., 2006; Tchokponhoué et al., 2020). Miraculin is a homodimeric protein obtained from the red berries of miracle fruit that exerts its taste-modifying activity by binding hT1R2–hT1R3 and functionally changing into an agonist at acidic pH (Koizumi et al., 2011). On the other hand, the extracts of the various tissues of the miracle fruit are of medicinal importance. For example, the extracts from the leaves show antidiabetic potential in type 2 diabetic rats, the stem has antioxidant bioconstituents that can inhibit human melanoma proliferation, and the extracts from fruit powder may be an effective treatment for acute gouty arthritis (Wang et al., 2011; Shi et al., 2016; Obafemi et al., 2017; Han et al., 2019; Huang et al., 2020). Moreover, miracle fruit also contains many important nutrients, such as proteins, lipids, vitamins, amino acids, and dietary phytochemicals (Njoku et al., 2015; He et al., 2016).

As an important group of tropical plants, there is no high-quality genome reported in the Sapotaceae family, which severely limits the utilization, breeding, and understanding of these species (Niu et al., 2020). Here, we report a high-quality and chromosome-level genome assembly of *S. dulcificum*, opening the gate for well-known of plants in the Sapotaceae family. The assembled genome size of *S. dulcificum* is ∼550 Mb, with contig N50 ∼14.14 Mb. We annotated 37,911 genes that were clustered into 15,799 gene families, and 3,828 and 4,739 gene families were expanded or contracted, respectively. Comparative genomic analysis revealed that miracle fruit underwent a whole-genome duplication (WGD) event, which had a close evolutionary relationship with *Camellia sinensis* and *Diospyros oleifera*. Moreover, metabolome and transcriptome analyses provide further information for understanding the mechanism of metabolite biosynthesis during fruit development. In addition, we found that the expression level of miraculin in the reproductive organs is very high, especially in the flesh of mature fruits with an fragments per kilobase of transcript per million (FPKM) value of ∼113,515, which is the highest expressed gene in the flesh. At the same time, we found that during the evolutionary process, histidine-30 residues and signal peptides were unique to miraculin in miracle fruit, which may have important implications for the production of its function and the evolution of which. We also found that the role of miraculin in miracle fruit is mainly to resist germ infection, defend against environmental pressure and regulate plant growth. As the first reference genome of *S. dulcificum*, this study will provide a valuable genomic resource for research on *S. dulcificum* and facilitate the production and utilization of *S. dulcificum*.

## Materials and Methods

### Plant Materials and Sequencing

The sequenced specimen of *S. dulcificum* was cultivated at Hainan University (20°N, 110°E) in Haikou, Hainan Province, China. Total genomic DNA was extracted from fresh leaves using the plant Genomic DNA Kit (TIANGEN, Beijing, China). The purified DNA was used as input material for the DNA library constructed and sequenced using the PacBio Sequel I and Illumina HiSeq 2,500 platforms at Novogene (Tianjing, China). Total RNA was extracted from root, stem, leaf, flower, flesh, and seed tissues from three developmental stages, including the young stage (green), turning stage (yellowish red), and mature stage (red), and three biological replicates of each tissue were set. RNA sequencing (RNA-seq) was then performed according to the standard process at Novogene (Tianjing, China).

### Genome Assembly and Quality Assessment

The *S. dulcificum* genome size was first assessed by flow cytometry (BD FACSCalibur) at OMIX Technologies Corporation (Chengdu, Sichuan, China). Karyotype analysis was performed to identify chromosome ploidy by fluorescence *in situ* hybridization (FISH) at OMIX Technologies Corporation (Chengdu, Sichuan, China). *K*-mer analysis (*K* = 19) was then used for heterozygosity and duplication rate assessment by GenomeScope (v2.0) (Ranallo-Benavidez et al., 2020) using paired-end Illumina data. Clean reads over 5k produced by PacBio sequencing were used for primary genome assembly by NextDenovo (v2.4.0).^1^ Preliminarily assembled contigs were polished by Pilon (v1.22) (Walker et al., 2014) using Illumina short reads. Finally, we mapped the high-quality Hi-C to the polished genome using Bowtie (v0.7.8) (Langmead and Salzberg, 2012), and a chromosome-level genome was generated using a 3D-DNA pipeline (180922) (Dudchenko et al., 2017). Benchmarking Universal Single-Copy Orthologs (BUSCOs) (v3.0.2, embryophyta_odb10) (Simao et al., 2015) analysis and the long-terminal repeat assembly index (LAI) (Ou et al., 2018) were used to assess assembly completeness. In addition, PacBio long reads, Illumina short reads, Hi-C data, and RNA-seq data were mapped to the assembly to calculate the coverage through BWA (0.7.17) (Li and Durbin, 2010) and Hisat2 (2.1.0) (Kim et al., 2015).

### Genome Annotation and Assessment

Homology-based prediction and *de novo* searching methods were employed to identify repetitive sequences in the *S. dulcificum* genome. RepeatModeler (v2.0.1) (Flynn et al., 2020) and LTR_Finder (v2.9.0) (Xu and Wang, 2007) were used to predict *de novo* repetitive sequences. Combining the *de novo* database and the known Repbase library, repetitive sequences were finally identified by using RepeatMasker (4.1.2-p1) (Tarailo-Graovac and Chen, 2009).

Protein-coding genes were predicted based on ab initio predictions, homology searching and RNA-seq data using the MAKER (v3.01.03) (Cantarel et al., 2008) pipeline. Augustus (v3.0.3) (Stanke et al., 2004) and SNAP (v2006-07-28) (Bromberg and Rost, 2007) were used for ab initio predictions. In homology searches, the protein sequences of *Arabidopsis thaliana*, *Actinidia chinensis*, *D. oleifera*, and *C. sinensis* were provided as protein evidence. For RNA evidence, the clean RNA-seq reads were assembled into inchworm contigs using Trinity (2.1.1) (Grabherr et al., 2011).

tRNAs were identified using tRNAscan-SE (v2.0.7) (Lowe and Eddy, 1997), rRNAs were detected using RNAmmer (v1.2) (Lagesen et al., 2007), and miRNAs and snRNAs were detected by searching the Rfam database (v12.2) (Kalvari et al., 2018) using infernal (v1.1.2) (Nawrocki and Eddy, 2013). The genome protein sequence of *S. dulcificum* was queried against the Plant Transcription Factor Database (PlantTFDB^2^) to identify transcription factors (TFs). The genome features (gene density, GC content, gene expression, repeat sequence density, and collinearity information) of *S. dulcificum* were visualized by Circos (v0.69) (Krzywinski et al., 2009).

### Gene Family Identification and Evolution Analysis

Protein sequences from the longest transcripts of each protein-coding gene in 11 species, including *Clethra arborea*, *Roridula gorgonias*, *A. chinensis*, *C. sinensis*, *D. oleifera*, *Aegiceras corniculatum*, *Vaccinium macrocarpon*, *A. thaliana*, *Vitis vinifera*, *Oryza sativa*, and *S. dulcificum*, were used to identify gene families using OrthoFinder (v2.3.11) (Emms and Kelly, 2015) based on an all-versus-all BLASTP (*e*-value ≤ 1e−5). The gene families of *D. oleifera*, *V. macrocarpon*, *A. corniculatum*, *A. chinensis*, and *S. dulcificum* were identified by OrthoVenn (v2.0) (Xu et al., 2019).

A total of 293 single-copy orthologous genes from the 11 species were obtained by OrthoFinder. The protein sequences of the 293 single-copy orthologous genes were aligned using Muscle (v3.8.31) (Edgar, 2004), and the results were used to construct a phylogenetic tree by FastTree (v2.1.10) (Price et al., 2010). The MCMCTree program in PAML (v4.9) (Yang, 2007) was used to estimate the divergence time among the 11 species combined with previously published calibration times [*A. thaliana* and *V. vinifera* diverged 107–135 million years ago (MYA) and *A. thaliana* and *O. sativa* diverged 115–308 MYA] in TimeTree^3^. The expansion and contraction of the gene families were calculated using CAFÉ (v4.2) (De Bie et al., 2006) based on gene family count, phylogenetic tree, and species divergence time. The Gene Ontology (GO) and Kyoto Encyclopedia of Genes and Genomes (KEGG) enrichment analyses were performed mainly using ClusterProfiler (v3.13) (Yu et al., 2012) and other in-house R scripts.

### Whole-Genome Duplication and Collinearity Analysis

To detect WGD events, the gene coding and protein sequences of *S. dulcificum*, *D. oleifera*, *C. sinensis*, and *A. thaliana* were used for synonymous substitution (Ks) value calculations by using KaKs_calculator (v2.0) (Zhang et al., 2006), and *A. thaliana* was used as a control. Homologous pairs were identified using an all-versus-all search in BLASTP (v2.5.0) (Altschul et al., 1990) and were used to identify syntenic blocks by WGD. The genome annotation information, coding sequence and protein sequence of *S. dulcificum* and *V. vinifera* were used as input files to perform synteny analysis through JCVI (v1.1.8)^4^. Dot plot analysis of *S. dulcificum* was produced by WGDI (v0.4.9) (Sun et al., 2021) using protein sequence and annotation information.

### Transcriptome and Metabolome Analysis

RNA sequencing clean data of the 30 samples mentioned above were mapped back to the *S. dulcificum* genome using Hisat2 (v2.1.0) (Kim et al., 2015), and then the results were processed by Samtools (v1.10-104-g869941a) (Li et al., 2009). The gene counts were calculated by HTSeq (v0.13.5) (Anders et al., 2015) and then normalized by StringTie (v2.1) (Pertea et al., 2015). Differentially expressed genes (DEGs) were then obtained by the DESeq2 (v1.32.0) (Love et al., 2014) package. GO and KEGG enrichment analyses were performed by ClusterProfiler. Weighted gene coexpression network analysis (WGCNA) was performed by the WGCNA (V1.70-3) (Langfelder and Horvath, 2008) package, and the soft threshold power was set to 10. The Pearson’s correlation coefficients (PCCs) of genes and metabolites were calculated using the Cor package.

For metabolite detection, the freeze-dried samples were first ground into powder by a grinder (MM 400 Retsch) at 30 Hz for 30 s. Then, 1.0 ml of 70% aqueous methanol was added to 100 mg of powder for metabolite extraction. The metabolites were quantified by the MRM method of LC–MS/MS 6500 (SCIEX, Boston, United States). The relative signal strengths of the metabolites were divided by intensities of the internal standard (0.1 mg/L lidocaine) for normalization (Chen et al., 2013). In the following analysis, log2 transformation was performed for the metabolite content values.

## Results

### Genome Sequencing and Assembly

**FIGURE 1:**
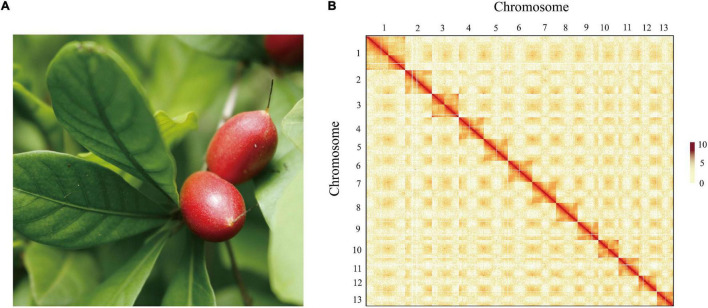
The morphology of the berry and Hi-C-assisted genome assembly of *S. dulcificum*. **(A)** The fruit of the sequenced *S. dulcificum*. **(B)** Hi-C interaction heatmap showing 110-kb resolution chromosome.

To obtain a high-quality chromosomal-level genome of *S. dulcificum* (Figure 1A), a combination of short-read Illumina sequencing, long-read PacBio sequencing, and the Hi-C sequence approach was applied. The genome size of *S. dulcificum* was first investigated with ∼565.60 Mb by flow cytometry (Supplementary Figure 1). In addition, we found that *S. dulcificum* is a diploid plant containing 2*n* = 2x = 26 chromosomes surveyed through FISH (Supplementary Figure 2). Relatively low heterozygosity (0.062%) and repetitive sequence content (37.5%) were found by *K*-mer analysis (*K* = 19; Supplementary Figure 3). A total of ∼119.57 Gb (226× coverage) of PacBio long reads was obtained, and reads longer than 5k were then used for the primary genome assembly. Subsequently, 104.90 Gb (∼198×) Illumina short reads were used for genome polishing, generating an improved version with a genome size of 568.98 M (contig N50 ∼14.14 M). We further scaffolded the genome to chromosome scale using ∼122.72 Gb (231× coverage) Hi-C clean data. Finally, we successfully clustered 63 contigs into 13 chromosomes by a 3D-DNA pipeline with an anchor ratio of ∼96.63% and generated a chromosome-level *S. dulcificum* genome (∼549.84 Mb) with chromosome lengths ranging from ∼29.30 Mb (Chr13) to ∼68.67 Mb (Chr1) (Figure 1B).

To assess the quality of the assembly, we mapped short reads from Illumina and Hi-C back to the final assembled genome, resulting in coverage of 99.29 and 93.64%, respectively. On the other hand, 96.56–98.27% of RNA-seq reads from 30 samples (see section “Materials and Methods”) were well aligned to the assembled genome (Supplementary Table 1). In addition, 1,326 (96.5%) of the 1,375 complete gene elements in the BUSCOs plant set were covered by the *S. dulcificum* genome (Supplementary Table 2). A relatively high LAI assessment score (19.152) indicates the high sequence continuity of assembly, which has met the standard of reference quality (Ou et al., 2018).

### Repeat Annotation and Gene Prediction

**FIGURE 2:**
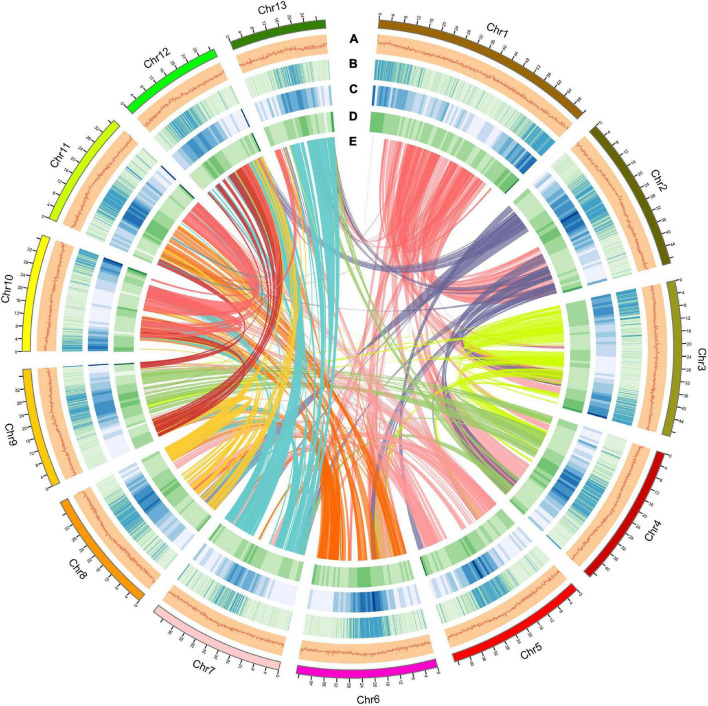
Overview of the *S. dulcificum* genome assembly. The outer layer of colored blocks is a circular representation of the 13 chromosomes, with thick mark labeling each 4 Mb. Layers a–e represent GC content distributions **(A)**, gene expression levels **(B)**, gene density **(C)**, repeat sequence density **(D)**, and inter-chromosomal synteny **(E)**, respectively. More deep mark labeling represents higher expression level, gene density, or repeat sequence density for **(B–D)**.

Using a combination of a *de novo* approach and a homology search, 305,010,596 bp (53.60%) of repetitive sequences were identified in *S. dulcificum*. Class I repeats (43.72%) were the main type; long terminal repeat (LTR) retrotransposons accounted for 40.20%. Gypsy and Copia, two main LTR-type transposons, accounted for 10.52 and 16.62% of the genome, respectively. Class II DNA transposons and unclassified repeats occupied 1.33 and 8.7% of the genome, respectively (Supplementary Table 3). For protein-coding gene prediction, a combination of ab initio, homolog, and transcriptome prediction strategies was used. In total, 37,911 protein-coding genes were predicted in *S. dulcificum*, with an average gene length of 3,951.5 bp, coding sequence length of 980.1 bp and exon number per gene of 4.12 (Table 1). The corresponding synteny along with GC content, gene expression levels, gene density, and distribution of TEs can be visualized in the CIRCOS diagram (Figure 2). In addition, non-coding RNA annotation yielded 117 microRNAs, 761 transfer RNAs, 215 ribosomal RNAs, and 94 small nuclear RNAs. In addition, 1,967 (5.18% in coding genes) TFs from 58 families were identified, and the largest TF families were bHLH, ERF, MYB, C2H2, and NAC (Supplementary Table 4). Furthermore, 33,725 protein-coding genes (88.95%) were supported by transcriptome data, and 30,296 protein-coding genes (81.37%) were assigned functions in at least one of the PFAM, COG, KEGG, or GO databases (Supplementary Table 5; Ashburner et al., 2000; Kanehisa and Goto, 2000; Mistry et al., 2020; Galperin et al., 2021).

**TABLE 1:** Statistics of *S. dulcificum* genome assembly and annotation.

Assembly feature	*Synsepalum dulcificum*
Number of contigs	81
Total size of contigs	569,013,336
Longest contig	39,932,979
Contig N50 count	13
Contig N50 length (bp)	14,129,605
Contig N90 count	40
Contig N90 length (bp)	5,031,503
GC content	33.80%
Chr count	13
Mean chr length (MB)	42.31
Number of protein-coding genes	37,911
Mean transcript length (bp)	3,951.5
Mean exon length (bp)	236.5
Mean exon per mRNA	4.12
Total repetitive sequences size (% of genome)	304,991,148 (53.60%)

### Specific, Expanded, and Contracted Gene Families in *Synsepalum dulcificum*

**FIGURE 3:**
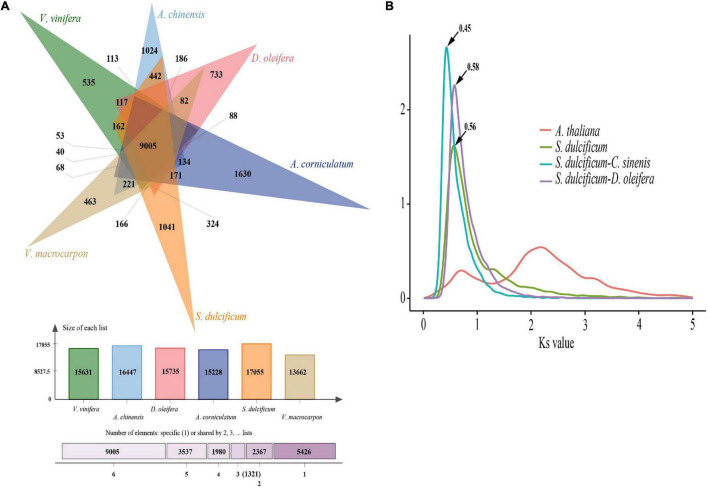
Gene family and WGD analysis of *S. dulcificum*. **(A)** Gene family distributions between *S. dulcificum*, *V. vinifera*, *A. chinensis*, *D. oleifera*, *V. macrocarpon*, and *A. corniculatum*. **(B)** Synonymous substitution rate (Ks) distributions of paralogs and orthologs of *A. thaliana*, *S. dulcificum*, *C. sinensis*, and *D. oleifera*.

As a special species in the Ericales order and Sapotaceae family, the special gene families of *S. dulcificum* may have unique functions. To identify the specific gene families in *S. dulcificum*, seven published genomes from Ericales, including *C. arborea* (Hartmann et al., 2020), *R. gorgonias* (Hartmann et al., 2020), *A. chinensis* (kiwi fruit) (Wu et al., 2019), *C. sinensis* (Chinese tea) (Xia et al., 2019, 2020), *D. oleifera Cheng* (wild kaki persimmon) (Zhu et al., 2019), *A. corniculatum* (Ma et al., 2021), and *V. macrocarpon* (American cranberry) (Diaz-Garcia et al., 2021); two representative eudicots [*A. thaliana* (Zapata et al., 2016) and *V. vinifera* (Jaillon et al., 2007)]; and one monocot [*O. sativa* (Sakai et al., 2013)] were chosen for gene family identification, in which *O. sativa* was chosen as the outgroup. A total of 32,771 (86.4%) genes in *S. dulcificum* were clustered into 15,799 gene families, leaving 5,140 genes that did not belong to any gene family (Supplementary Table 6). To clearly show the unique gene families of *S. dulcificum*, five important species (*V. vinifera*, *A. chinensis*, *D. oleifera*, *V. macrocarpon*, and *A. corniculatum*) were selected for further analysis. The results showed that 9,005 gene families were shared among miracle and other species, and 1,041 gene families were found to be specific to miracle fruit (Figure 3A and Supplementary Table 6). GO and KEGG enrichment analyses revealed that these specific gene families were mainly enriched in “sesquiterpene biosynthetic process,” “phytoalexin biosynthetic process,” “monoterpenoid biosynthesis,” and “brassinosteroid biosynthesis” (Supplementary Figures 4, 5 and Supplementary Table 7). In addition, a total of 18,640 gene families were inferred in the most recent common ancestor (MRCA) from the above 11 species by analyzing gene family expansion and contraction. In *S. dulcificum*, 3,828 gene families expanded, and 4,739 gene families contracted. GO and KEGG enrichment analysis of 102 significantly expanded gene families revealed a marked enrichment in genes involved in “PPAR signaling pathway,” “phenylpropanoid biosynthesis,” “defense response to fungus,” “secondary metabolite biosynthetic process,” and “pigment biosynthetic process” (Supplementary Figures 6, 7 and Supplementary Table 8).

### Phylogenetic Placement of *Synsepalum dulcificum* as a Sister to *Camellia sinensis* and *Diospyros oleifera*

**FIGURE 4:**
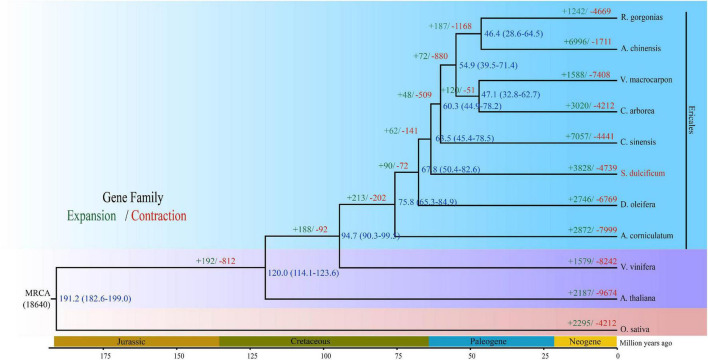
Phylogenetic tree of *S. dulcificum* and 10 other species. The time of species differentiation and the expansion and contraction of gene families are denoted at each node. Green and red font numbers represent expansion and contraction of gene families, blue font words are species differentiation time.

The Ericales order belongs to the Asterids clade, which together with Rosids consists of two main clades of Eudicots. To date, a total of 22 species from eight families have been sequenced in Ericales.^5^ Regarding the loss of high-quality genome information from the Sapotaceae family, the phylogenetic position is uncertain. To investigate the phylogenetic placement of *S. dulcificum*, we constructed a phylogenetic tree based on 293 strictly single-copy ortholog gene sets from the 11 species above used for gene family analysis. The results show that *S. dulcificum* is closest to *C. sinensis* and *D. oleifera*, indicating that Sapotaceae family plants are much closer to species of Theaceae and Ebenaceae. The high-confidence phylogenetic tree and calibration points selected from the TimeTree website reflect that the divergence time between *S. dulcificum* and *C. sinensis* is ∼63.5 (45.4–78.5) MYA, and that between *S. dulcificum* and *D. oleifera* is ∼67.8 (50.4–82.6) MYA (Figure 4).

### Whole-Genome Duplication and Collinearity Analysis of *Synsepalum dulcificum*

**FIGURE 5:**
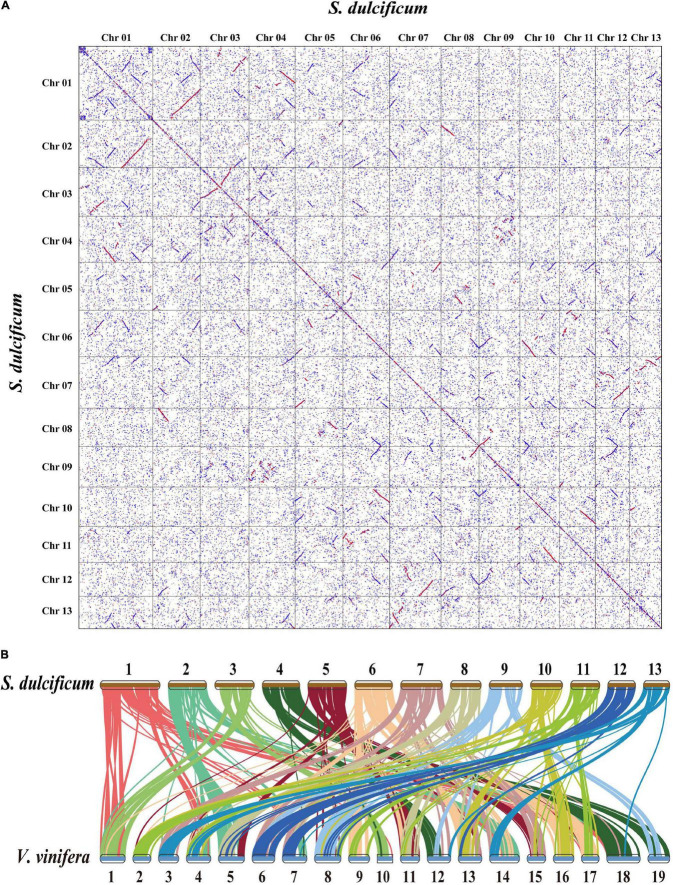
Genomic synteny analysis between chromosomes of *S. dulcificum*, and between *S. dulcificum* and *V. vinifera*. **(A)** Dot plots of paralogs in *S. dulcificum* genome. **(B)** Collinear relationships between *S. dulcificum* and *V. vinifera*.

Whole-genome duplication events are important driving forces of plant evolution. To assess the WGD events of *S. dulcificum*, we selected a range of species (*D. oleifera*, *C. sinensis*, and *A. thaliana*) to calculate the synonymous substitution rate (Ks) values with duplicate gene pairs. A major peak of *S. dulcificum* was detected at Ks = 0.56 (Figure 3B), indicating that *S. dulcificum* underwent a WGD event. The peak value (Ks = 0.45) of orthologs between *S. dulcificum* and *C. sinensis* was lower than the value of *S. dulcificum* (Ks = 0.56) itself, indicating that speciation between *S. dulcificum* and *C. sinensis* occurred later, and the *S. dulcificum* showed one-to-one syntenic relationship with *C. sinensis* (Supplementary Figure 8), which suggested that *C. sinensis* might share a common WGD event with *S. dulcificum* (Figure 3B). The peak value (Ks = 0.58) of orthologs between *S. dulcificum* and *D. oleifera* was larger than that of *S. dulcificum*, implying that they diverged earlier and that the WGD event occurred soon after they diverged, which corresponds to the phylogenetic relationship in Figure 4. The synteny dot plot analysis reveals that duplications within *S. dulcificum* are either interchromosomal (between chromosomes 1 and 2, 1 and 4, 2 and 8, 5 and 8, 6 and 10, 7 and 12, 7 and 13, 8 and 9, and 10 and 11) or intrachromosomal (Chr1 and Chr3) duplications (Figure 5A). The intergenomic collinearity between *S. dulcificum* and *V. vinifera* indicated that the *S. dulcificum* genome has a two-to-one syntenic collinearity relationship with *V. vinifera*, which also proved that *S. dulcificum* underwent a WGD event after the eudicot ancestor evolved (Figure 5B).

### Metabolite and Transcript Profiles During Fruit Development Stages

**FIGURE 6:**
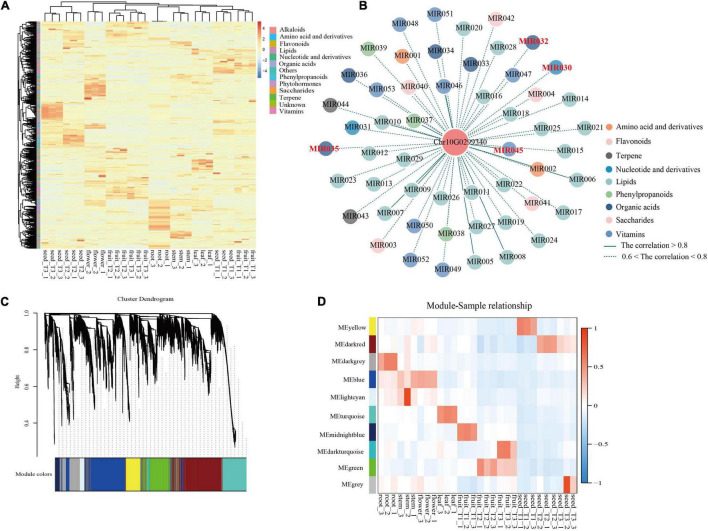
Integrated analysis of metabolome and transcriptome revealed the functions of miraculin in *S. dulcificum*. **(A)** Heatmap of metabolites detected in six tissues including fleshes and seeds from three stages of *S. dulcificum* fruits. **(B)** Regulatory network of metabolites highly related to miraculin. **(C)** The dendrogram of co-expression modules identified by WGCNA across samples. **(D)** The heatmap of the correlation between samples and modules. [MIR030, inosine 5′-monophosphate; MIR032, citric acid; MIR035, urate; MIR045, pyridoxine (vitamin B6)].

Comprehensive metabolic profiling of roots, stems, leaves, flowers, flesh, and seeds from three developmental stages [T1, young fruit (green); T2, turning-stage fruit (yellowish red); T3, mature fruit (bright red)] of *S. dulcificum* was performed by widely targeted metabolite analysis based on LC–MS/MS. A total of 855 metabolites were identified, with 697 annotated metabolites including 203 lipids, 137 vitamins, 100 amino acid derivatives, 73 organic acids, 63 flavonoids, 36 phenylpropanoids, 28 terpenes, 25 nucleotides and derivatives, 19 saccharides, 6 alkaloids, and 6 phytohormones (Supplementary Table 12 and Supplementary Figure 9). A heatmap with all metabolites showed a good correlation among the three biological replicates of all samples (Figure 6A). The metabolite compositions between samples were clearly separated in the PCA diagram, indicating the spatiotemporal specificity of metabolites in miracle fruit (Supplementary Figure 10).

To study the changes in metabolites during fruit development, we compared the differences in metabolite content among T3, T2, and T1. Metabolic analysis found that 117 metabolites had higher levels in T2 than in T1, and 63 metabolites had higher levels in T3 than in T2. These increased metabolites were mainly lipids, vitamins, amino acids, and their derivatives (Supplementary Table 13). The increased metabolites were subjected to enrichment analysis using the KEGG database. The results of T2 vs. T1 showed that these enriched metabolic pathways were mainly “biosynthesis of plant hormones,” “vitamin B6 metabolism,” “ubiquinone and other terpenoid-quinone biosynthesis,” “glycosylphosphatidylinositol (GPI)-anchor biosynthesis,” etc. (Supplementary Table 14). The T3 vs. T2 results showed that these enriched metabolic pathways were mainly “phenylpropanoid biosynthesis,” “phenylalanine metabolism,” “glutathione metabolism,” “biosynthesis of phenylpropanoids,” etc. (Supplementary Table 15). We found that 32 metabolic pathways including “anthocyanin biosynthesis,” “biosynthesis of terpenoids and steroids,” “phenylpropanoid biosynthesis,” and other pathways were enriched in both T2 vs. T1 and T3 vs. T2. This result indicates that these metabolites involved in the common metabolic pathway may play a more important role during fruit development.

RNA sequencing data were generated for six different tissues and three different periods, which correspond to the metabolome samples. We obtained 8.11 Gb of high-quality clean reads on average after original data filtration. The three biological replicates of each sample were found to have a good correlation by calculating the expression levels indicated by FPKM reads (Supplementary Figure 11). In addition, 28,560 genes were found to be expressed (FPKM ≥1) in 30 samples. The PCA also supported the classification of the gene expression heatmap (Supplementary Figure 12).

To gain further insight into the regulation of the transcriptome dynamic changes throughout fruit development, DEG analysis was performed among T3, T2, and T1 fruit samples. A total of 9,246 and 3,524 DEGs were identified in T2 vs. T1 and T3 vs. T2, respectively, based on *p*-value < 0.05 and | log2Fold Change | ≥ 1. Among them, 3,824 DEGs were upregulated and 5,418 DEGs were downregulated in T2 vs. T1; in addition, 2,092 DEGs were upregulated and 1,432 DEGs were downregulated in T3 vs. T2. To investigate the specific functions of the upregulated DEGs, GO, and KEGG analyses were performed for T2 vs. T1 and T3 vs. T2. “Phenylpropanoid biosynthetic process,” “cellular response to chitin,” “flavonoid biosynthetic process,” and 388 other GO terms were significantly enriched in T2 vs. T1. “Glucosinolate metabolic process,” “lanosterol synthase activity,” “response to brassinosteroid,” and 358 other GO terms were significantly enriched in T3 vs. T2 (Supplementary Figures 13, 14 and Supplementary Table 16). “Amino sugar and nucleotide sugar metabolism,” “fatty acid biosynthesis,” “biosynthesis of secondary metabolites,” and 118 other metabolic pathways were enriched in T2 vs. T1. “Plant–pathogen interaction,” “MAPK signaling pathway,” “plant hormone signal transduction,” and 107 other metabolic pathways were enriched in T3 vs. T2 (Supplementary Figures 15, 16 and Supplementary Table 17). By comparing the KEGG pathways of the two comparisons (T3 vs. T2 and T2 vs. T1), we found that differential metabolites and DEGs were enriched in the same pathways, such as “steroid biosynthesis,” “vitamin B6 metabolism,” and “flavonoid biosynthesis” (Supplementary Table 18). These enriched pathways suggest that metabolite changes may be positively regulated by genes and provide insights into the genetic basis of metabolic processes underlying different fruit development stages in *S. dulcificum*.

On the other hand, PCCs between miraculin and metabolites were also calculated, and 57 metabolites were found to be highly correlated (PCC >0.6) with miraculin (Figure 6B and Supplementary Table 19). Among them, there are many metabolites that are particularly important for plant growth and defense. For example, citric acid (MIR032) is involved in fruit growth, vitamin B6 (MIR045) is involved in heat stress, urate (MIR035) is involved in responses to the pathogenic fungus, and inosine 5′-monophosphate (MIR030) is involved in salt stress.

### The Evolution and Function of Miraculin

**FIGURE 7:**
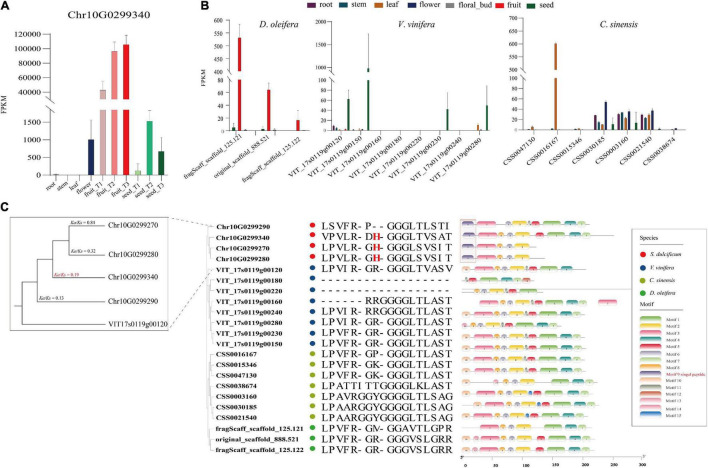
Expression level, phylogenetic tree, and unique protein sites of miraculin and its homologous genes in *S. dulcificum* and genetically close species. **(A)** Expression levels of miraculin in various tissues of *S. dulcificum*. FPKM values were used. **(B)** The expression level of homologous genes of miraculin in tissues of *D. oleifera*, *V. vinifera*, and *C. sinensis*. **(C)** The phylogenetic tree, selective pressure analysis, and protein sequence alignment of miraculin and its homologous genes in *S. dulcificum*, *V. vinifera*, *C. sinensis*, and *D. oleifera*.

As the most special protein in miracle fruit, miraculin has been well studied in terms of structure, taste-modifying activity mechanisms, subcellular localization, etc., but few reports have focused on its evolution and function in miracle fruit itself (Paladino et al., 2008; Takai et al., 2013; Sanematsu et al., 2016). In this study, an interesting phenomenon was found: the miraculin gene (Chr10G0299340) was the most highly expressed gene in fruit flesh, with an FPKM value of ∼113,515, which is consistent to the report of high protein level of miraculin in the fruit of *S. dulcificum* (Hiwasa-Tanase et al., 2012; Figure 7A). To investigate the peculiar properties of miraculin, three homologous genes of miraculin from *S. dulcificum*, seven from *C. sinensis*, three from *D. oleifera*, and eight from *V. vinifera* were obtained. The expression levels of these homologous genes were determined by a standard RNA-seq analysis process using published data (see Supplementary Table 9). The results showed that the expression levels of miraculin in the fruit of *S. dulcificum* were significantly higher than the expression of the homologous genes in other species (Figure 7B). Furthermore, a phylogenetic tree was constructed using the protein sequences of these homologous genes. Obviously, the phylogenetic tree divided into four clades in accordance with the species, indicating the relative species specificity of the miraculin family (Figure 7C). Ka/Ks analysis showed that the miraculin gene has undergone purifying selection (Figure 7C). In addition, we also found that the signal peptide motif (motif 9) of miraculin is unique in *S. dulcificum* and has been reported to function in the transfer of miraculin outside the plasma membrane (Takai et al., 2013; Figure 7C). It has been reported that the histidine-30 residue is the key point for the taste-modifying activity of miraculin (Ito et al., 2007). Compared with the homologous genes from other species, the histidine-30 residue is a unique site in *S. dulcificum*. Besides, we compared the gene expression (Supplementary Figure 17), protein sequence and functional annotation of the miraculin homologous genes in *S. dulcificum*, the results showed that only Chr10G0299340 has a higher expression level in mature fruits. Thus, we speculated that the extremely high expression level in the flesh of fruit, the unique signal peptides, and the histidine-30 residue together form the specific characteristics of miraculin in *S. dulcificum*.

To dissect the function of miraculin in *S. dulcificum*, WGCNA was performed to investigate the coexpression networks of miraculin. A total of 19,850 genes were screened out after discarding the low expression genes (FPKM <10), which were then used to construct coexpression network modules, resulting in 10 modules (Figure 6C). We found that miraculin was located in the yellow module, which contained 2,647 genes (Figure 6D and Supplementary Table 10). PCCs between genes from the yellow module were calculated, genes highly related to miraculin (PCCs >0.6) were obtained, and 331 genes remained. GO analysis showed that the terms “chitinase activity,” “response to fungus,” “seed germination,” “seed maturation,” and other terms related to resistance to adversity were significantly enriched (Supplementary Figure 18 and Supplementary Table 11). The KEGG analysis showed that the pathways “metabolic pathways,” “oxidative phosphorylation,” and “biosynthesis of secondary metabolites” were significantly enriched (Supplementary Table 11).

## Discussion

*Synsepalum dulcificum* originating from western and central Africa is a rare plant distributed in tropical and subtropical regions. In this research, we constructed a high-quality chromosome-level reference genome of *S. dulcificum* by combining Illumina short-read data, PacBio long-read data and Hi-C data. The assembly genome size is ∼550 Mb, with a contig N50 of 14.14 Mb. A total of 94.3% complete BUSCOs present in the *S. dulcificum* assembly indicated high genome integrity. In addition, 37,911 protein-coding genes were predicted by a combination of ab initio, homolog, and transcriptome prediction strategies. This is the first chromosome-level reference genome of the Sapotaceae family, which provides important genomic data for *S. dulcificum* studies and provides a reference for studies of other species in the Sapotaceae family.

In this study, 15,799 gene families were identified, 1,041 of which were specific for *S. dulcificum*. Enrichment analysis found that these specific gene families were involved in many important processes, such as “sesquiterpene biosynthetic process,” “phytoalexin biosynthetic process,” and “monoterpenoid biosynthesis.” Phylogenetic analysis based on 293 single-copy orthologous genes revealed that *S. dulcificum* was most closely related to *C. sinensis* and *D. oleifera*, which provides new insight into the evolutionary relationship of species in the Ericales order. The divergence time between *S. dulcificum* and *C. sinensis* was ∼63.5 MYA, and that between *S. dulcificum* and *D. oleifera* was ∼67.8 MYA. The divergence time between the *D. oleifera* and *C. sinensis* lineages in previous reports is ∼85.3 and ∼75 MYA, which is slightly larger than that in our study (Akagi et al., 2020). We speculate that this may be because we used more genome data from species of the order Ericales, especially the genome data of *S. dulcificum* of the Sapotaceae family that we constructed in our study. We also found that 3,828 gene families had expanded and that 4,739 gene families had contracted in the *S. dulcificum* genome, which were mostly involved in the “DNA replication proteins,” “PPAR signaling pathway,” “defense response to fungus,” etc.

As one of the important driving forces for plant evolution, a WGD event was found in *S. dulcificum*. In addition, the *S. dulcificum* genome has good collinearity with *V. vinifera*. However, whether the WGD that occurred in *S. dulcificum* is shared with other species in the Ericales order remains unclear. Previous studies have provided evidence that *C. sinensis* experienced only WGD events after the core-eudicot WGT event, and WGD was shared by at least 17 families in Ericales (Wang et al., 2021). Ebenaceae, Theaceae, and Actinidiaceae may share the same WGD event, which occurred ∼110 MYA. Combined with the evolutionary position of *S. dulcificum* in the order Ericales (Zhang et al., 2020), we speculate that the WGD of *S. dulcificum* may also be shared with other species of Ericales but is not specific to the Sapotaceae family. Interestingly, *C. sinensis* and *D. oleifera* have 15 chromosomes, but *S. dulcificum* has only 13 chromosomes. The ancestral chromosomal base number of the core order Ericales is believed to be 9 (Soza et al., 2019). Thus, research on the evolutionary history of *S. dulcificum* would be meaningful and important for species in the Sapotaceae family, even in Ericales.

Furthermore, we also performed metabolite detection and transcriptome sequencing from six tissues of miracle fruit, including three stages of fruit flesh and seeds. A total of 697 annotated metabolites were detected, and 28,560 genes were expressed. Metabolite difference analysis found that the contents of lipids, vitamins, amino acids, and their derivatives increased during fruit development. The KEGG enrichment analysis of the increased metabolites showed that these pathways were enriched in many important processes. We also performed GO and KEGG enrichment analysis for upregulated DEGs in T3 vs. T2 and T2 vs. T1. The significantly enriched GO terms and KEGG pathways were mainly “phenylpropanoid biosynthetic process,” “cellular response to chitin,” “sugar metabolism,” “fatty acid biosynthesis,” “biosynthesis of secondary metabolites,” etc. We found that many metabolites and DEGs were enriched in the same pathway, which provides insights for understanding the molecular mechanism of important metabolite biosynthesis.

In this study, an interesting phenomenon was found: the miraculin gene (Chr10G0299340) was the most highly expressed gene in fruit flesh, with an FPKM value of ∼113,515, indicating the potential high protein level of miraculin in *S. dulcificum*. To investigate the peculiar properties of miraculin, three homologous genes of miraculin from *C. sinensis*, *D. oleifera*, and *V. vinifera* were identified. The gene expression of Chr10G0299340 is at least 100 times that of its homologous genes, and we found that it has signal peptides and histidine-30 residues that other homologous genes do not have. Thus, we speculated that the extremely high expression level in the flesh of fruit, the unique signal peptides, and the histidine-30 residue together form the specific characteristics of miraculin in *S. dulcificum*. In previous studies, researchers were only concerned about the benefits of miraculin to the human body and never studied the function of miraculin on the miracle fruit itself (Koizumi et al., 2011; Swamy et al., 2014; Huang et al., 2020). In our research, combining WGCNA, enrichment analysis and metabolite correlation analysis, we believed that miraculin mainly plays a role in regulating seed germination and maturation, resisting pathogen infection, resisting environmental pressure, and regulating plant growth, which is consistent with what has been reported regarding the function of miraculin-like proteins in grape (Ohkura et al., 2018), coffee (Mondego et al., 2011), tomato (Brenner et al., 1998), and rough lemon (Tsukuda et al., 2006). The above results indicated that the peculiar property of miraculin that modifies sour tastes to sweet tastes may be collateral, and the main meaning of its existence is to benefit itself.

In summary, the high-quality reference genome sequence, metabolomic and transcriptomic data of different tissues and periods of *S. dulcificum* obtained in our study provide valuable resources for both functional genomic research and genetic improvement breeding of miracle as well as other economically important plants in the Sapotaceae family.

## Data Availability Statement

All sequencing data of the genome have been deposited into the CNGB sequence archive of CNGBdb with accession number CNP0002330 (https://db.cngb.org/). And the sequencing data of RNA-seq have been deposited in the NCBI Sequence Read Archive database under accession number PRJNA778426.

## Author Contributions

YD and HH designed and supervised the project. ZL, HX, YC, PD, PL, and WL prepared the samples. ZY, ZL, and HX analyzed the data. ZY wrote the manuscript. JL, YD, and HH revised the manuscript. All authors read and approved the final manuscript.

## Conflict of Interest

The authors declare that the research was conducted in the absence of any commercial or financial relationships that could be construed as a potential conflict of interest.

## Publisher’s Note

All claims expressed in this article are solely those of the authors and do not necessarily represent those of their affiliated organizations, or those of the publisher, the editors and the reviewers. Any product that may be evaluated in this article, or claim that may be made by its manufacturer, is not guaranteed or endorsed by the publisher.

## Footnotes

1
https://github.com/Nextomics/NextDenovo

2
http://planttfdb.gao-lab.org/prediction.php

3
http://www.timetree.org

4
https://zenodo.org/record/31631#.YUvM4LgzaUk

5
https://www.plabipd.de/index.ep
