# Supplementary material for: The Chromosome-Level Genome of Miracle Fruit (Synsepalum dulcificum) Provides New Insights Into the Evolution and Function of Miraculin
Source: Front Plant Sci. 2022 Jan 3;12:804662. doi: 10.3389/fpls.2021.804662 (PMC8763355; doi:10.3389/fpls.2021.804662)
Supplement: Supplementary file 1 [file Data_Sheet_1.PDF]

Supplementary Fig. 1

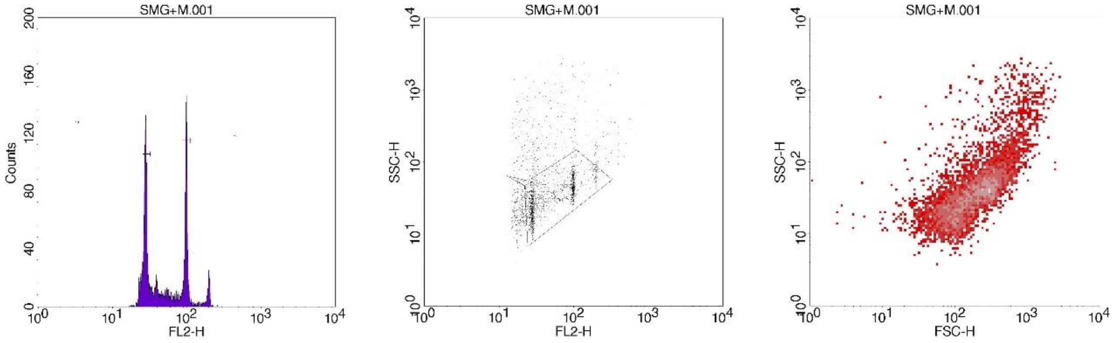

Sample ID	Internal Species	Fluorescence stronger (Internal control)	Fluorescence stronger Sample	Ratio	Genome size (G)	Genome size average	Stdev
	Zea mays	96.78	27.6	0.29	0.66		
S. dulcificum	*Zea mays*	98.87	27.43	0.28	0.64	0.65	0.01
	Zea mays	96.18	27.3	0.28	0.65		

Supplementary Fig. 1 The histogram of flow cytometry analyses of *S. dulcificum*.

Zea mays was used as an internal reference in Flow cytometry analyses, and 3 replications were set. Finally, the genome size of *S. dulcificum* was estimated nearly 0.65Gb by comparing to the fluorescence stronger of internal control.

Supplementary Fig. 2

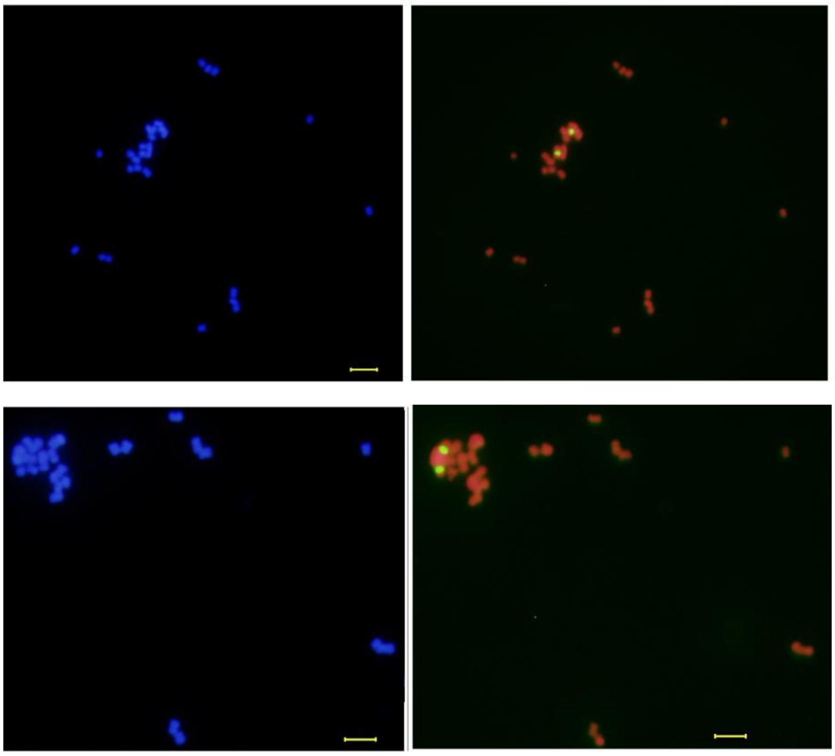

Supplementary Fig. 2: Fluorescence in situ hybridization (FISH) showed the chromosome number and ploidy of *S. dulcificum*.

FISH was performed by using the root of *S. dulcificum*, and 5S rDNA was used as fluorescence probe (green), the scale is 5 microns. The chromosome number and ploidy are 2n=2x=26 according to FISH observation.

Supplementary Fig. 3

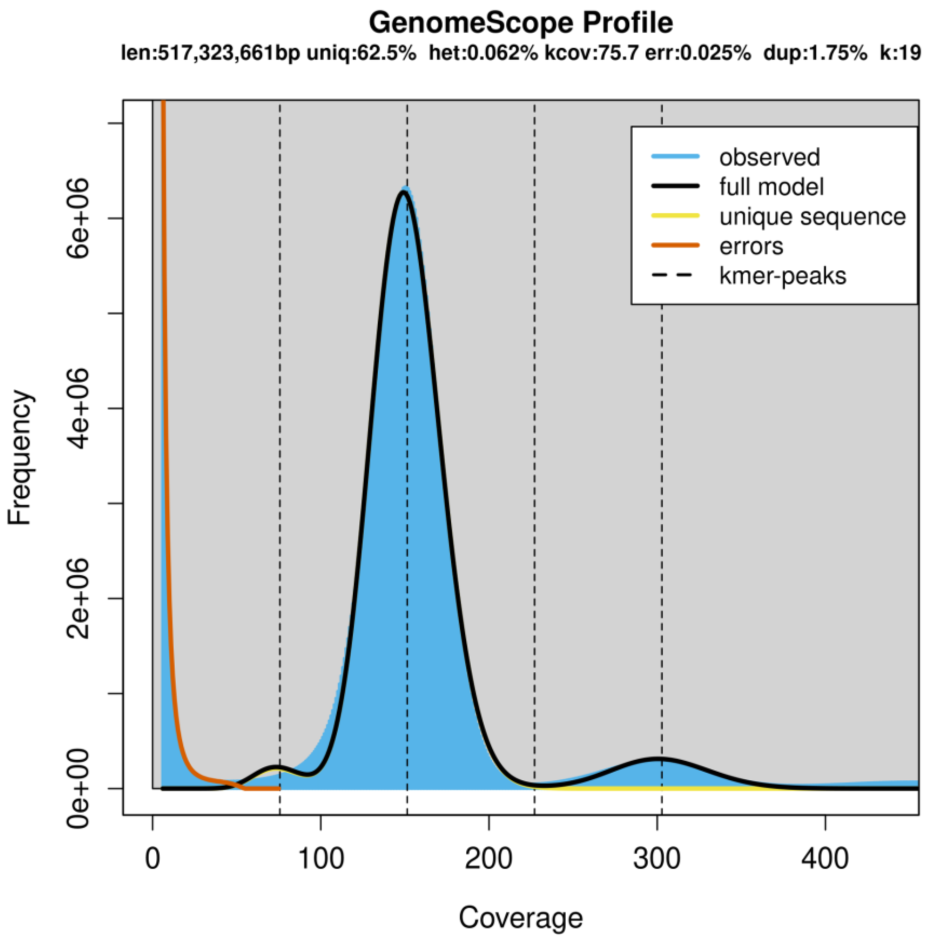

Supplementary Fig. 3: Genome size, heterozygous and duplications of *S. dulcificum* predicted by K-mer (k=19) analysis.

K-mer distribution was caculated by jellyfish, and GenomeScope was used to draw the photograph. The genome size was estimated 517.32 Mb, genome heterozygosity was 0.062% and genome duplication was 1.75%.

Supplementary Fig. 4

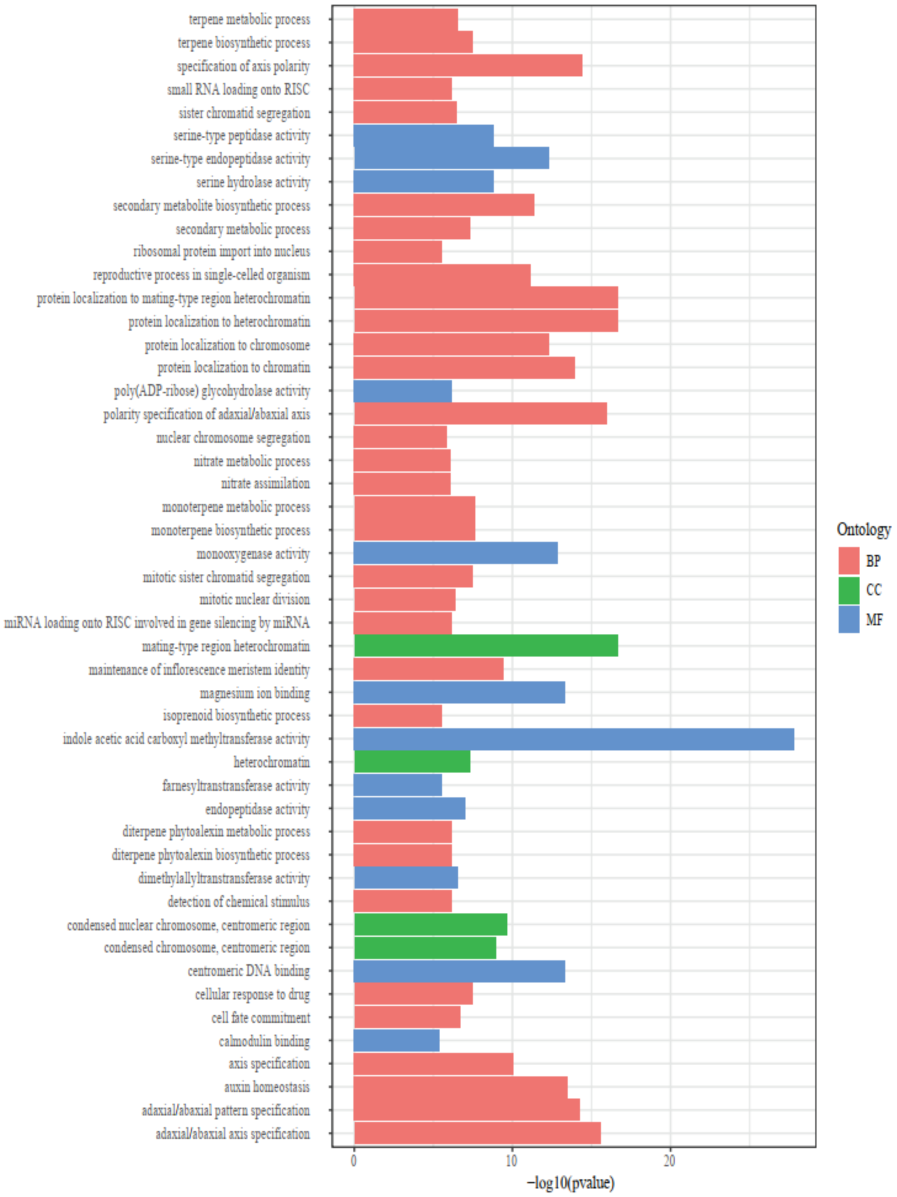

Supplementary Fig. 4: GO enrichment of the genes from unique families of *S. dulcificum*.

BP: Biological Process. CC: Cellular Components. MF: Molecular Function.

Supplementary Fig. 5

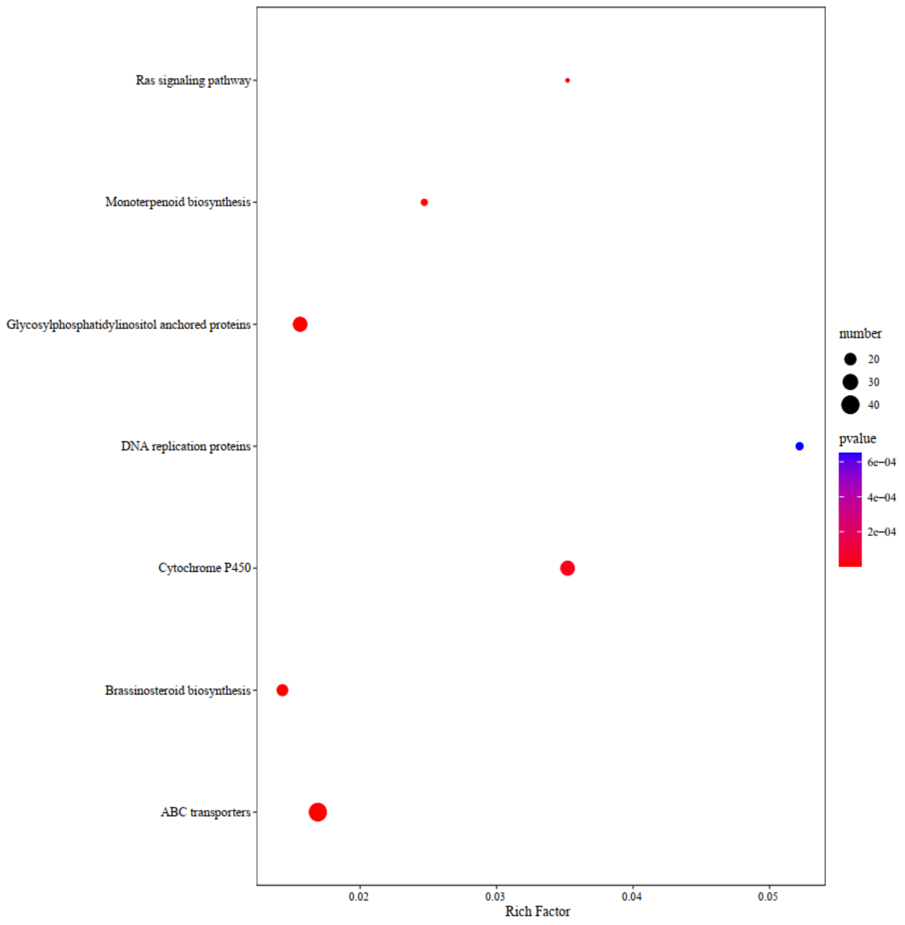

Supplementary Fig. 5: KEGG enrichment of the genes from unique families of *S. dulcificum*.

Supplementary Fig. 6

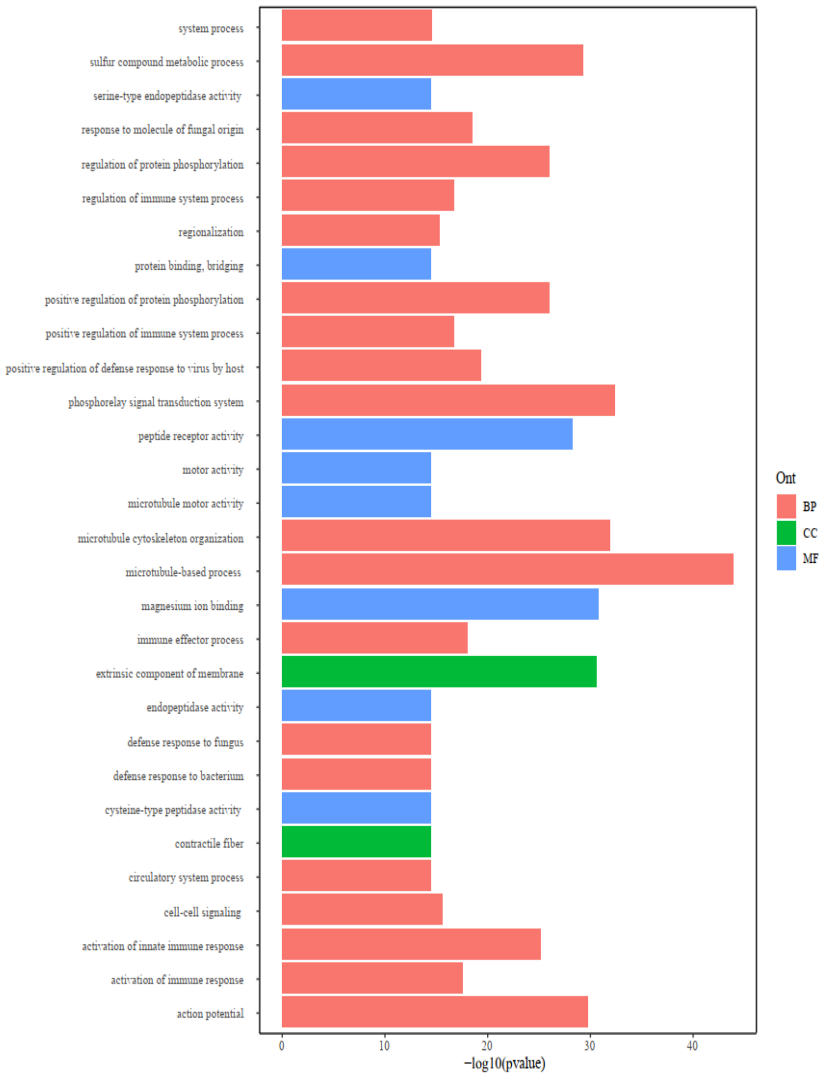

Supplementary Fig. 6: GO enrichment of the genes in the expanded families of *S. dulcificum*.

Supplementary Fig. 7

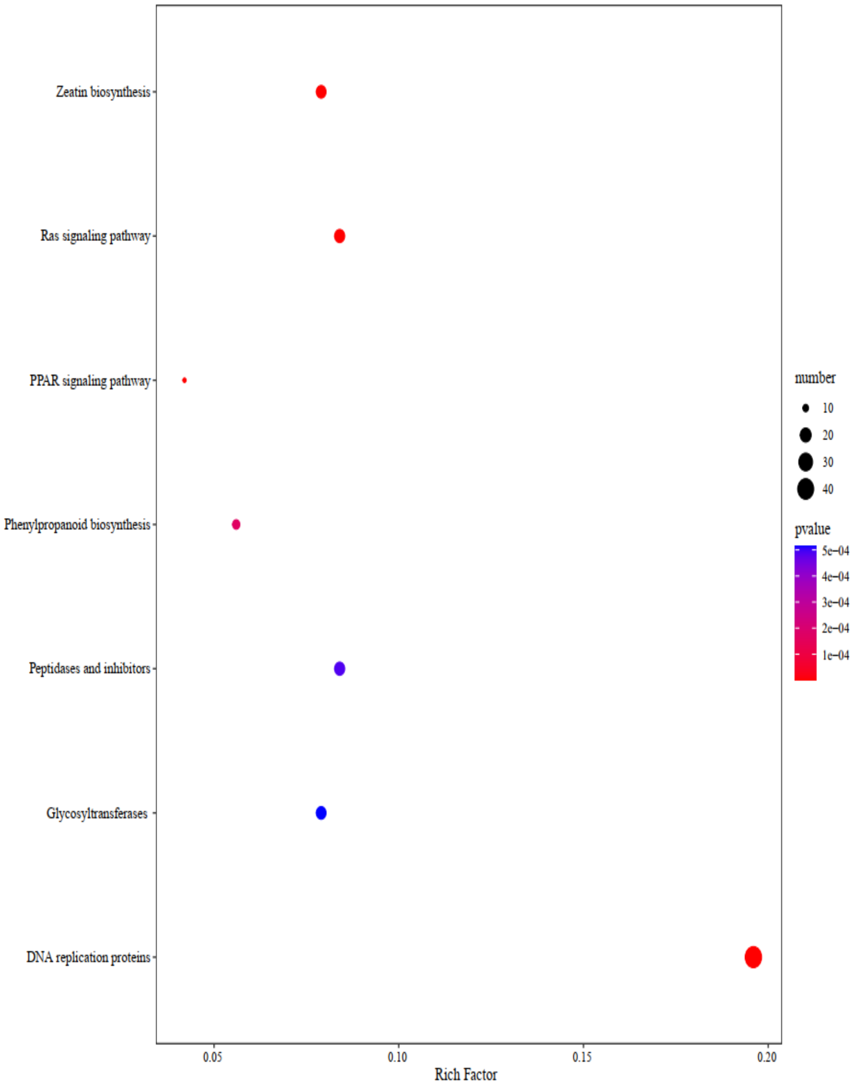

Supplementary Fig. 7: KEGG enrichment of the genes in the expanded families of *S. dulcificum*.

Supplementary Fig. 8

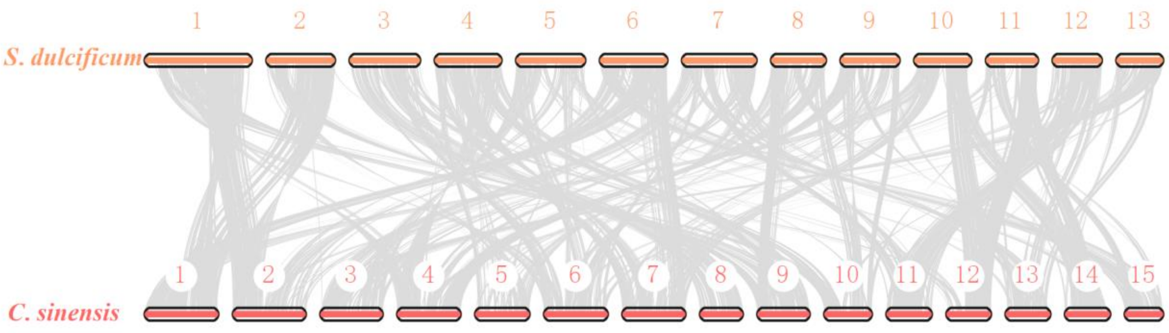

Supplementary Fig. 8: Genomic synteny analysis between of *S. dulcificum* and *C. sinensis*.

Supplementary Fig. 9

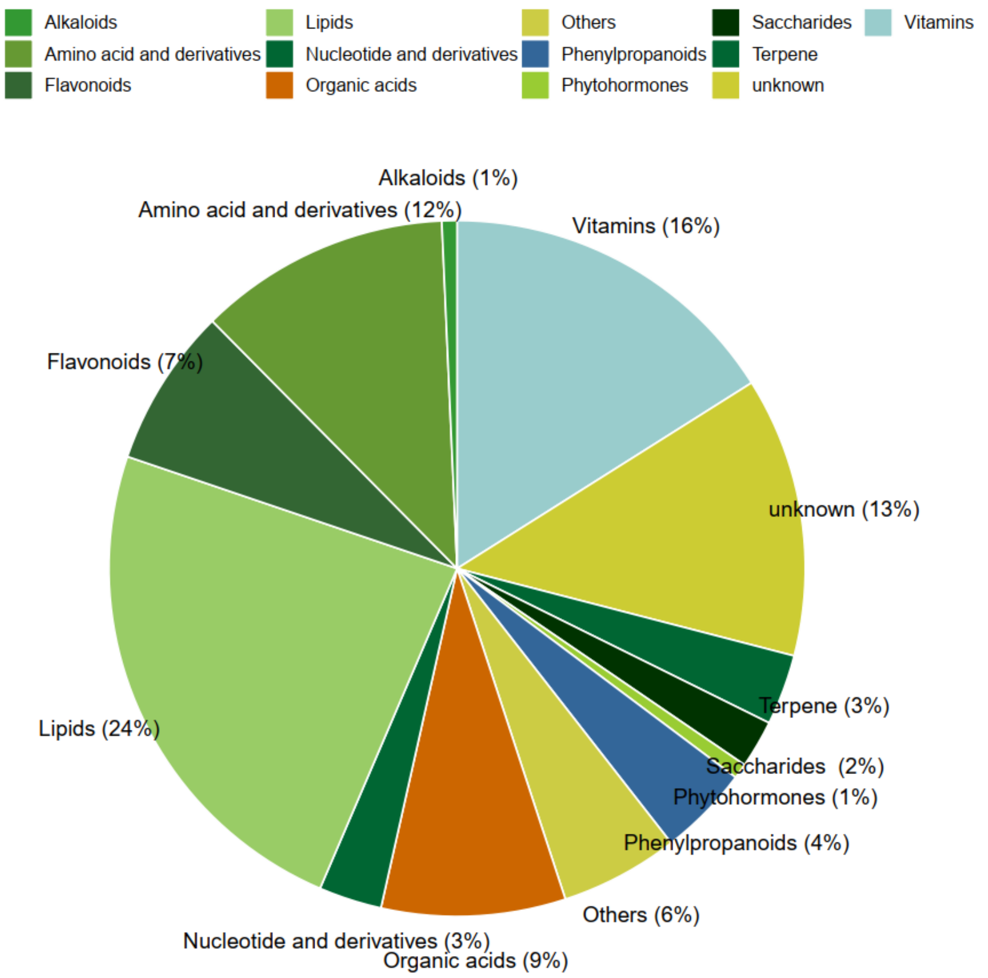

Supplementary Fig. 9: The pie figure of classification of all metabolites detected in 6 tissues including fleshes and seeds of 3 different stages of fruit from *S. dulcificum*.

Supplementary Fig. 10

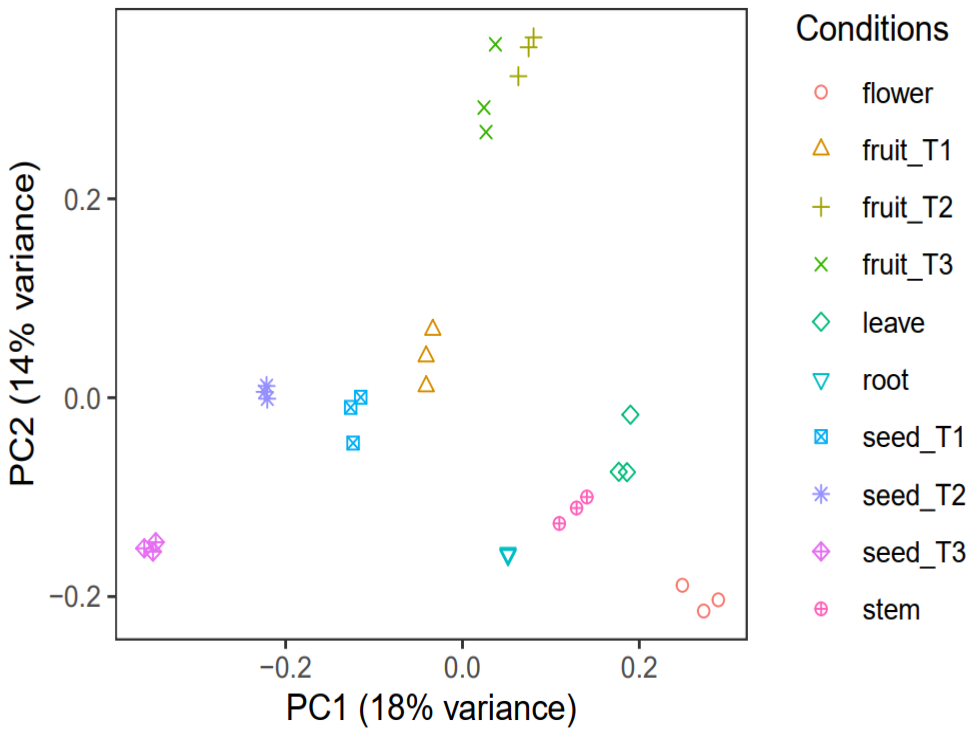

Supplementary Fig. 10: Principal component analysis (PCA) of all metabolome from samples of *S. dulcificum*.

A total of 30 samples from 6 tissues including fleshes and seeds from 3 different stages were used. Three biological replications for each tissue were set.

Supplementary Fig. 11

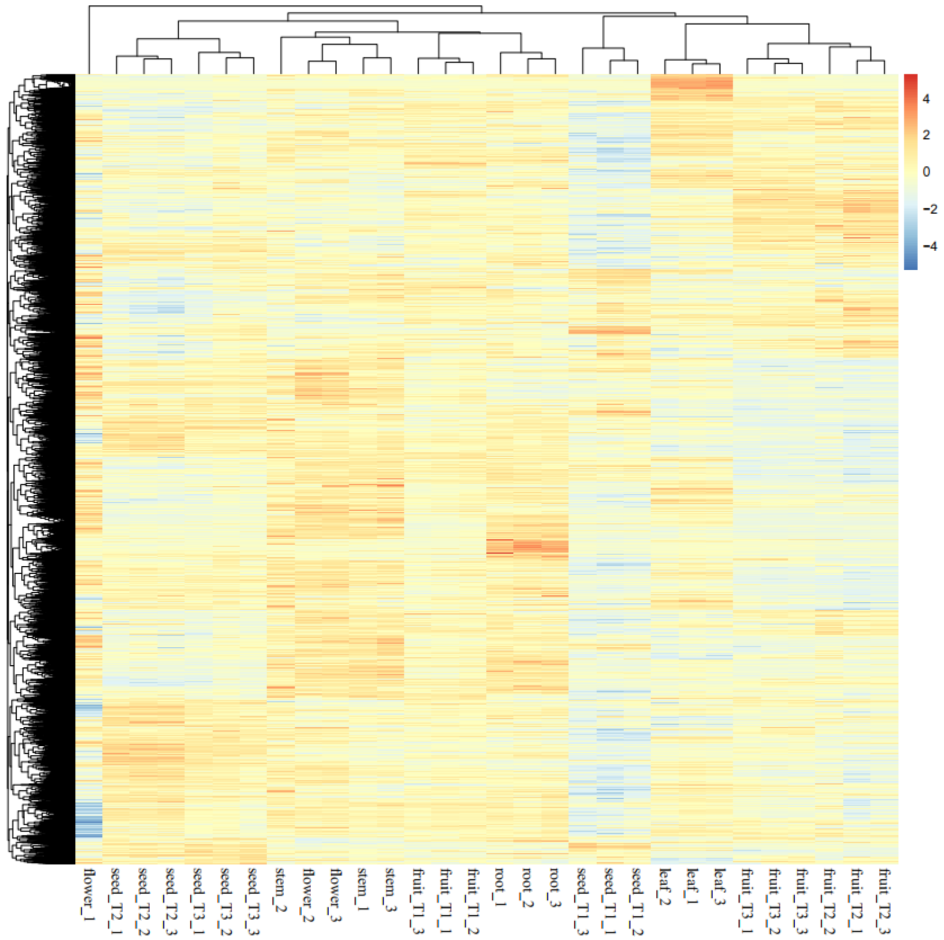

Supplementary Fig. 11: The heat map of all identified genes from RNA-seq data in 6 tissues including fleshes and seeds from 3 different stages of *S. dulcificum*.

Supplementary Fig. 12

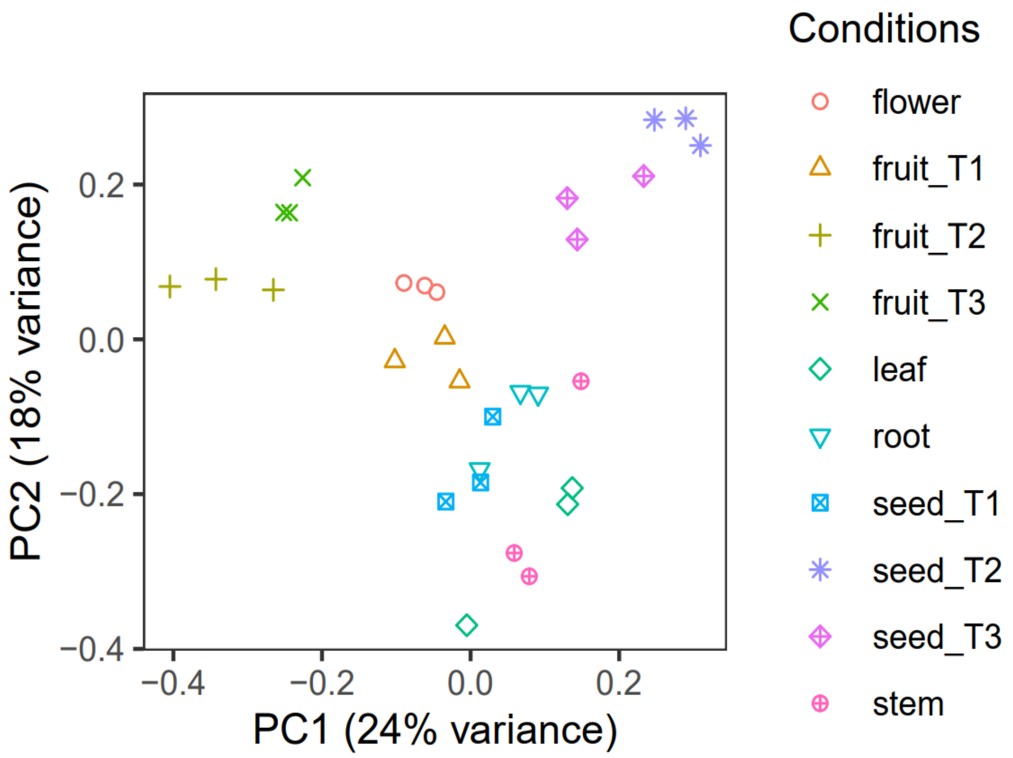

Supplementary Fig. 12: PCA of all RNA-seq samples with 3 biological replications of *S. dulcificum*.

Supplementary Fig. 13

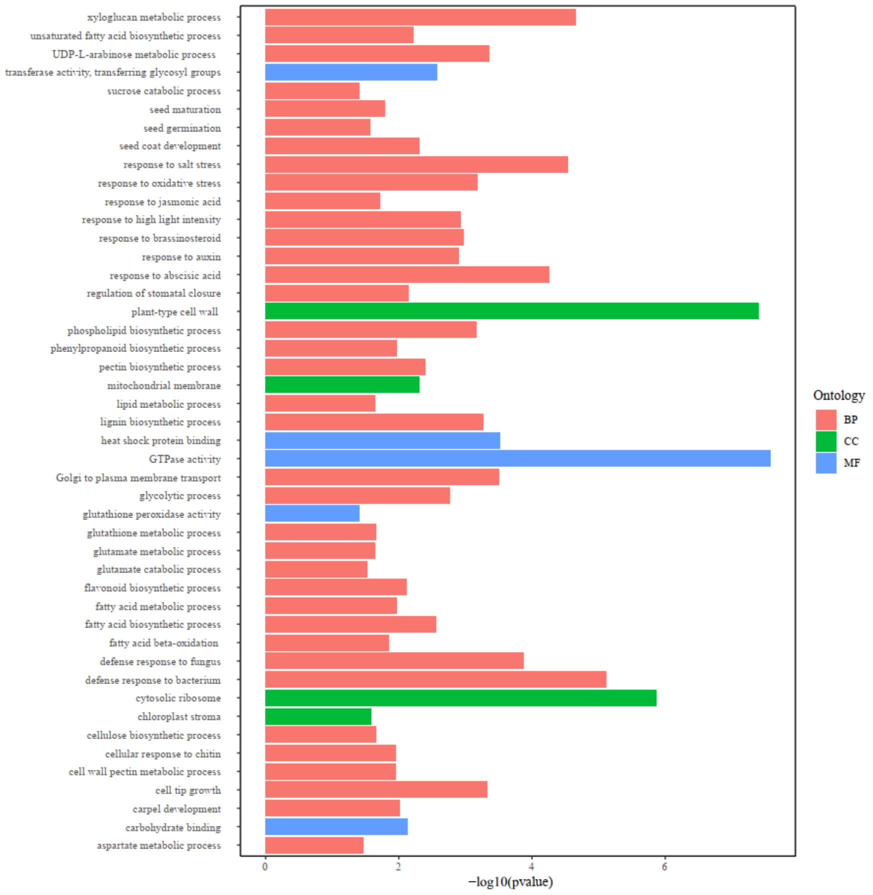

Supplementary Fig. 13: GO enrichment of DEGs up-regulated in T2 vs T1.

Supplementary Fig. 14

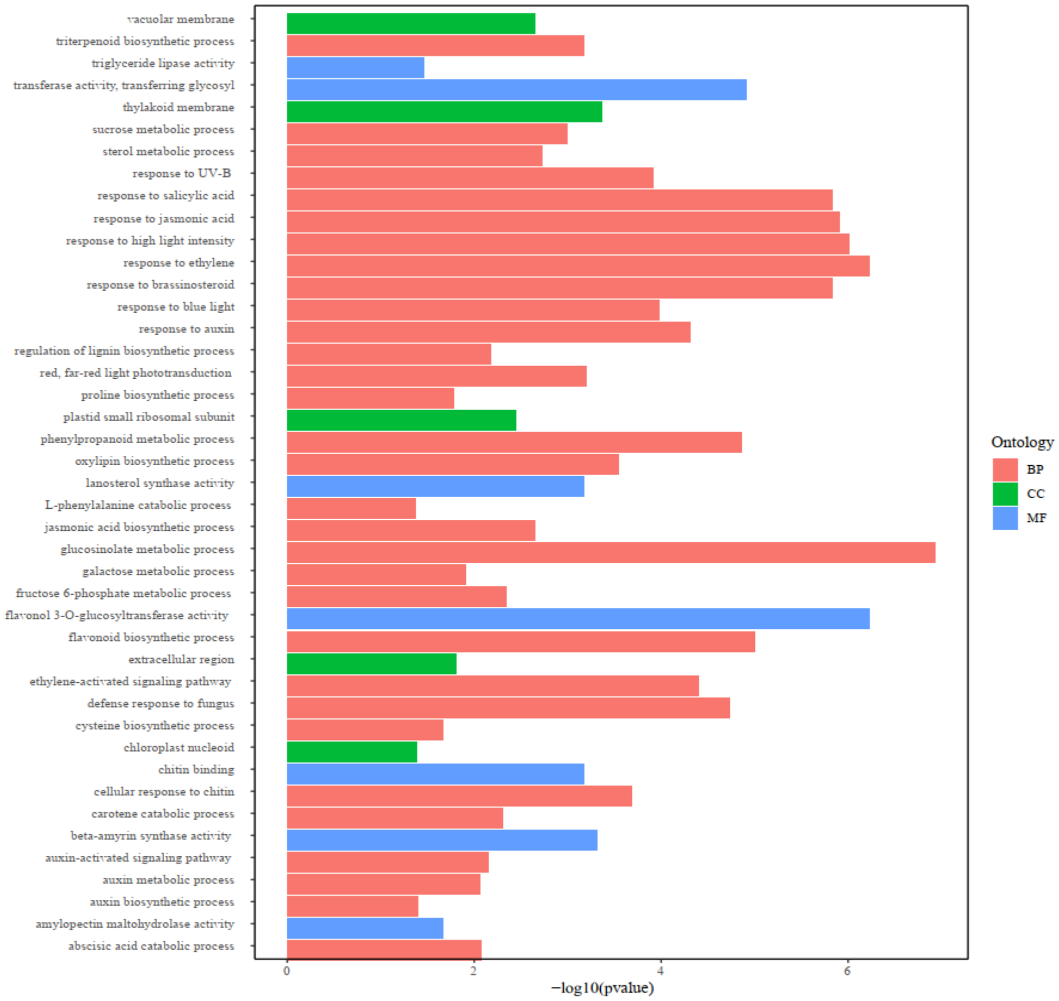

Supplementary Fig. 14: GO enrichment of DEGs up-regulated in T3 vs T2.

Supplementary Fig. 15

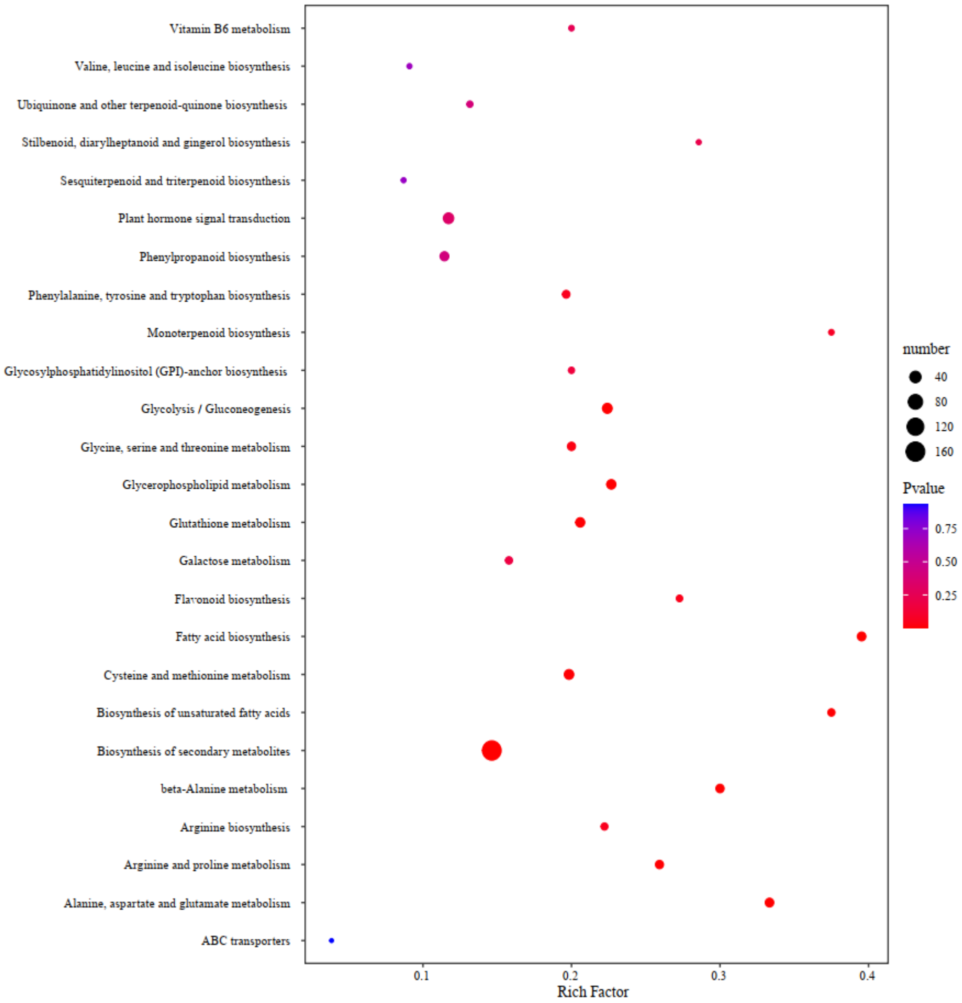

Supplementary Fig. 15: KEGG enrichment of DEGs up-regulated in T2vsT1.

Supplementary Fig. 16

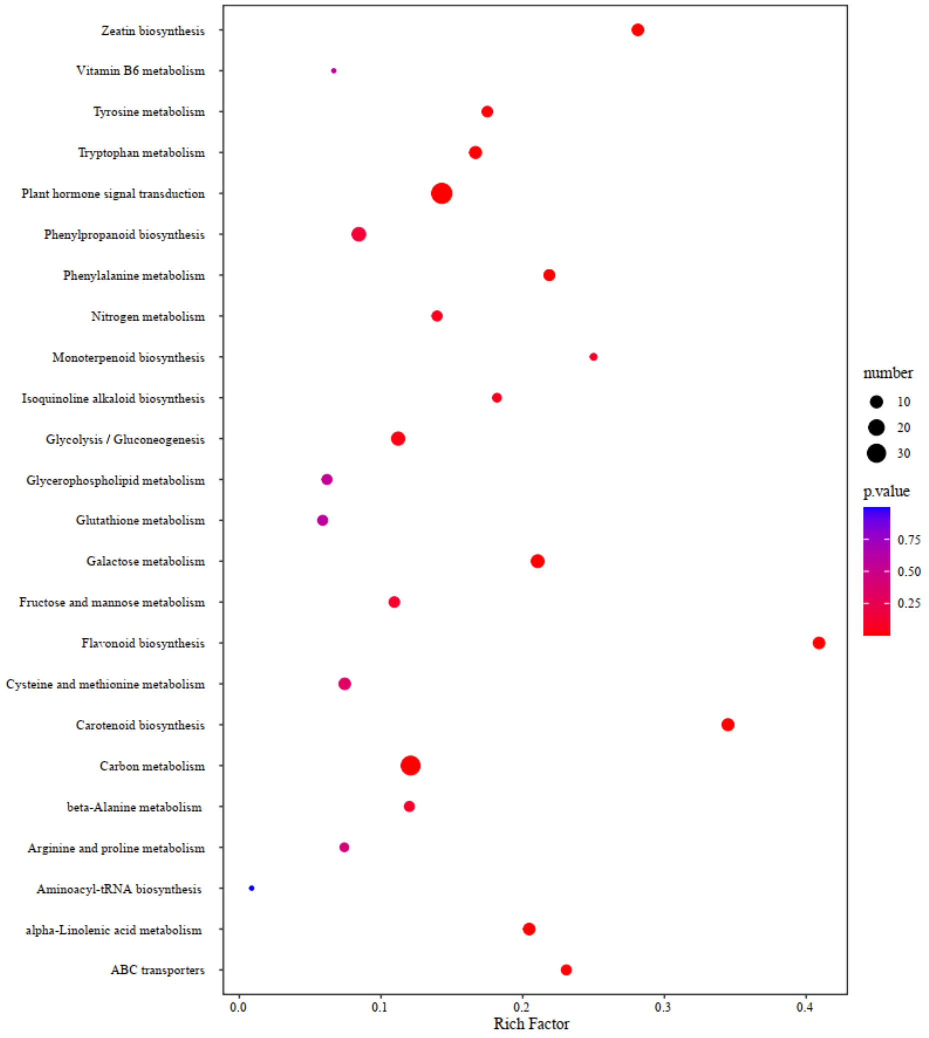

Supplementary Fig. 16: KEGG enrichment of DEGs up-regulated in T3 vs T2.

Supplementary Fig. 17

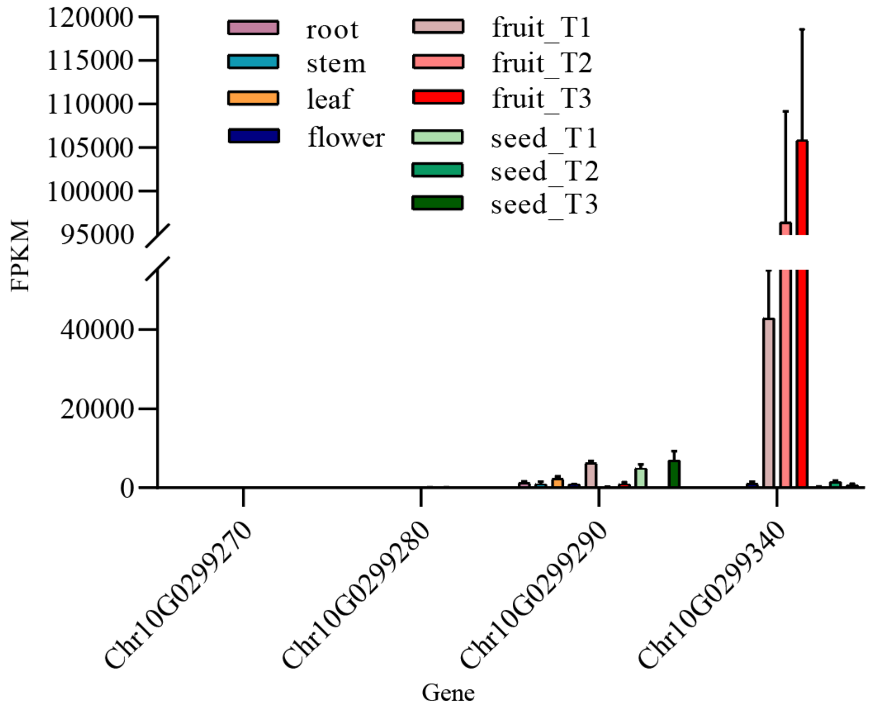

Supplementary Fig. 17: The expression level of miraculin homologous genes in S. dulcificum.

Supplementary Fig. 18

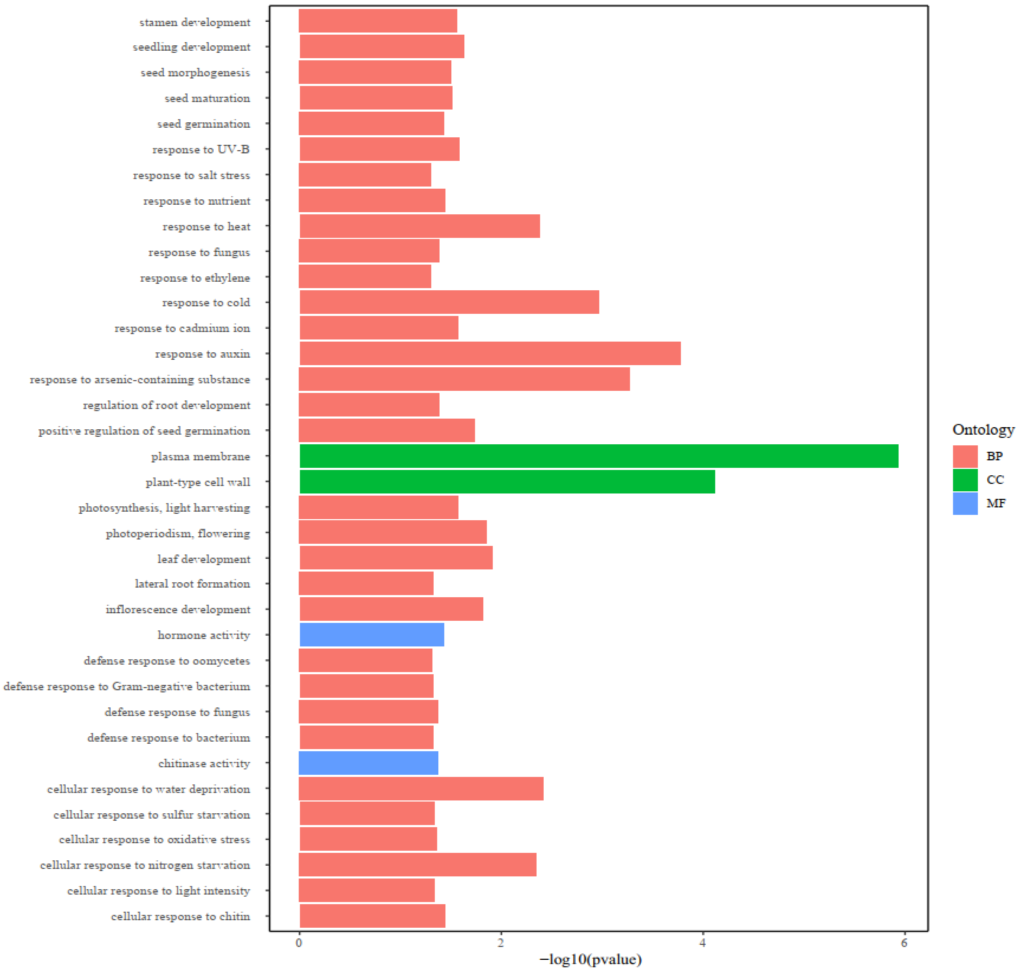

Supplementary Fig. 18: GO enrichment of genes highly related to the miraculin (PCCs > 0.6).
